# Unusual Presentation of Thoracic Epidural Ewing Sarcoma in a 20-Year-Old Patient: A Case Report

**DOI:** 10.7759/cureus.62902

**Published:** 2024-06-22

**Authors:** Daniel Markov, Kristian I Bechev, Galabin Markov, Simona D Hadzhieva, Vladimir Aleksiev, Elena G Poryazova

**Affiliations:** 1 General and Clinical Pathology, Medical University of Plovdiv, Plovdiv, BGR; 2 Neurological Surgery, University Multidisciplinary Hospital for Active Treatment (UMHAT) Pulmed, Plovdiv, BGR; 3 Medicine, Medical University of Plovdiv, Plovdiv, BGR; 4 Neurology, University Hospital "Pulmed", Plovdiv, BGR; 5 Cardiovascular Surgery, Medical University of Plovdiv, Plovdiv, BGR

**Keywords:** oncological treatment, pathohistological aspects, surgical treatment, spine, ewing's sarcoma

## Abstract

The aim of this case report is to present a rare case of epidural Ewing sarcoma with spinal cord compression, which is an uncommon presentation of this tumor.

Ewing's sarcoma is a primary malignant tumor predominantly affecting individuals in their second decade of life, primarily impacting those aged 10 to 25, with the average age of onset being around 20 years. Epidemiological studies reveal that this cancer most commonly arises in the diaphyses of the long tubular bones in the lower extremities. Spinal involvement, however, is exceedingly uncommon.

A case of sacral type of Ewing's sarcoma, with the most common localization of the primary spinal sarcomas and an extremely aggressive course, has been described in the literature. Other localizations of Ewing's sarcoma located in other areas of the spine are also presented. Even rarer are cases in which the tumor formation is located epidurally and exhibits marked medullary compression and absent neurological symptoms.

We present the case of a 20-year-old patient who was admitted to the neurology department with symptoms of lower flaccid paraparesis and pelvic-reservoir dysfunction, specifically urinary retention for 16-17 hours, after which a catheter was added. MRI revealed an epidural tumor spanning TH5-TH7 vertebral levels, causing significant spinal cord compression. A CT scan of the chest identified a tumor on the left side at the level of the sixth rib, featuring soft tissue involvement, rib destruction, lung invasion, and a small pleural effusion.

Due to the critical neurological symptoms, the patient underwent emergency surgery in the neurosurgical department, which included thoracic laminectomies, maximal possible tumor resection, and effective spinal cord decompression. Postoperative period was uneventful. Histopathological examination confirmed the diagnosis of Ewing's epidural sarcoma. The patient subsequently received adjuvant chemotherapy and radiotherapy. Six months post-treatment, the patient demonstrated a satisfactory overall condition with significant improvement in gait and continues to undergo chemotherapy courses.

## Introduction

For the first time, James Ewing in 1921 introduced the term diffuse malignant bone endothelioma, which is a highly malignant primary malignant tumor occurring at a young age, typically in the second decade of life [1]. In this way, Ewing's sarcoma primarily localizes in the diaphyses of the long tubular bones of the limbs. It is much less frequently found in the spine, with a preference for the sacral bone, and extremely rarely occurs in the epidural space of the spinal canal. Primitive neuroectodermal tumor (pNET) and Ewing's sarcoma (ES) are part of the same spectrum of primary malignancies, which also include malignant small cell tumor of the chest wall (Askin's tumor). Primary ESs are considered histologically as undifferentiated small-cell tumors [2]. Literature data indicate a prevalence of 3.5-14.9% spinal involvement from all other locations [3].

We report a case of a 20-year-old patient with the clinic of lower flaccid paraparesis, with moderate severity and retention disturbances of the pelvic reservoirs. Due to the progressively worsening neurological status and the performed imaging diagnosis of the thoracic spine showing Ewing's epidural sarcoma, as part of the multidisciplinary treatment, operative decompression, by means of laminectomy followed by maximum tumor excision, played a primary role.

What stands out in this case is the location of Ewing's sarcoma in the epidural space in the thoracic region.

## Case presentation

Clinical features

A 20-year-old male patient was admitted to the neurology department due to gradually progressive symmetrical muscle weakness and numbness in the legs, lasting for two days, beginning at the feet and ascending to the torso, eventually leading to complete immobility and difficulty urinating for 16-17 hours. The patient's medical history was unremarkable, with no recent infectious diseases, alcohol or substance use or smoking. Additionally, there were no reports of appetite loss or weight loss.

On neurological examination, the patient exhibited severe lower flaccid paraparesis (Grade III) in both lower limbs, muscle hypotonia, and markedly diminished knee reflexes to absent Achilles reflexes bilaterally. Babinski signs were positive bilaterally, and there was conductor-type hypesthesia at the TH8 dermatome level, urinary retention, and polyneuritis syndrome.

**Figure 1:**
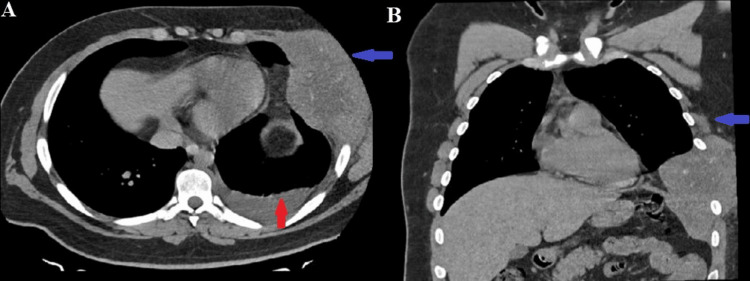
(A and B). A native CT scan of the lung in the axial and coronal plane shows a tumor formation (Ewing's sarcoma) located on the left in the region of the 6th rib, along the mid-axillary line, with the presence of a soft tissue lesion, destruction of the adjacent rib. Propagation to the lung, with infiltration of the pleura and chest wall, dimensions 15-10 cm (blue arrow). A small pleural effusion on the left (red arrow).

**Figure 2:**
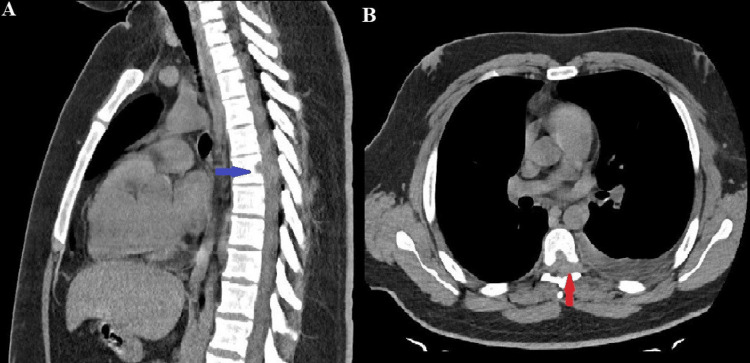
(A and B). A segment of a native CT scan of the thoracic spine in the sagittal and axial planes. Figure 2A shows the destruction of the body of TH7 vertebra and prolapse of the isodense formation towards the dural sac (blue arrow). Figure 2B presents an axial section through the level of the TH7 vertebra and the presence of an isodense formation filling the posterolateral (left) epidural space, with the border between the spinal cord and the epidural space obliterated (red arrow).

CT scan of the chest and thoracic compartments was performed, which revealed a soft tissue formation in the area of ​​the 6th rib on the left along the middle axillary line with destruction of the same and a small reactive pleural effusion. Two oval parenchymal metastases are present on the right side in the region of the 5th and 6th lung segments, suggesting possible secondary involvement from the primary malignancy (Figure 1A, 1B). Destruction of the TH7 vertebral body was noted, with the presence of a small epidural tumor mass at the levels of TH5 to TH7 vertebral projections, with spinal medullary compression (Figure 2A, 2B).

**Figure 3:**
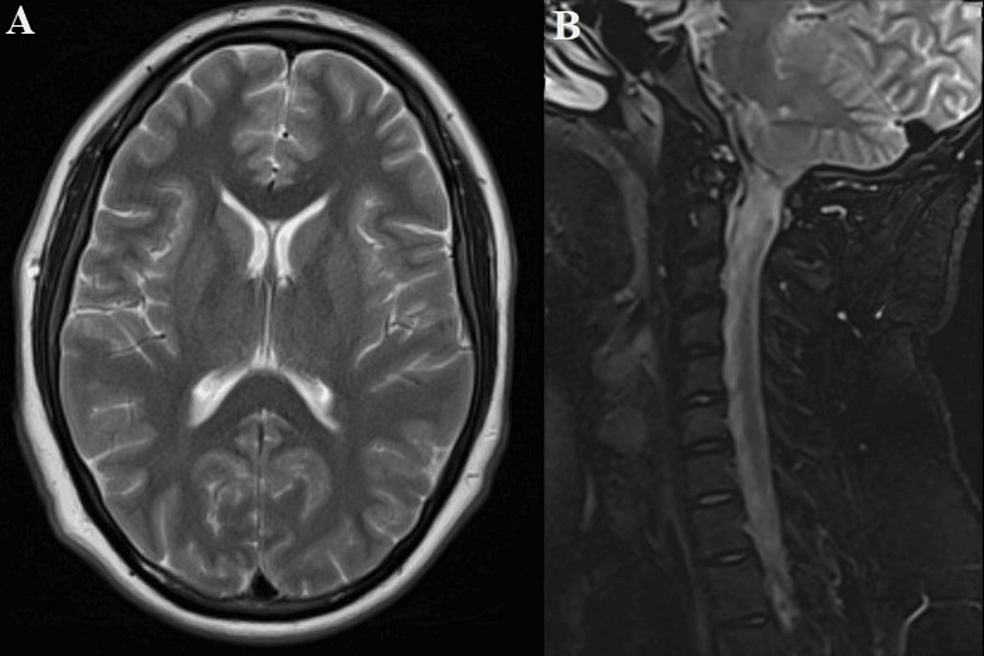
(A and B). MRI of the cranio-spinal transition. Figure 3A shows an axial section of the brain at the level of the lateral ventricles and thalamic structures, showing no abnormal imaging findings. Figure 3B shows a sagittal section of the cervical myelon where no MS plaques were detected.

A lumbar puncture was performed with evidence of protein-cell dissociation. In a differential-diagnostic plan, considerations were directed to Guillain-Barre syndrome, given the progressively developing weakness in the lower limbs, tendon hyporeflexia to areflexia, and protein-cell dissociation, but the electromyography conducted did not meet the criteria for this disease. An MRI of the brain and posterior cranial fossa was performed, which did not show changes directly correlated with the clinical complaints (Figure 3).

**Figure 4:**
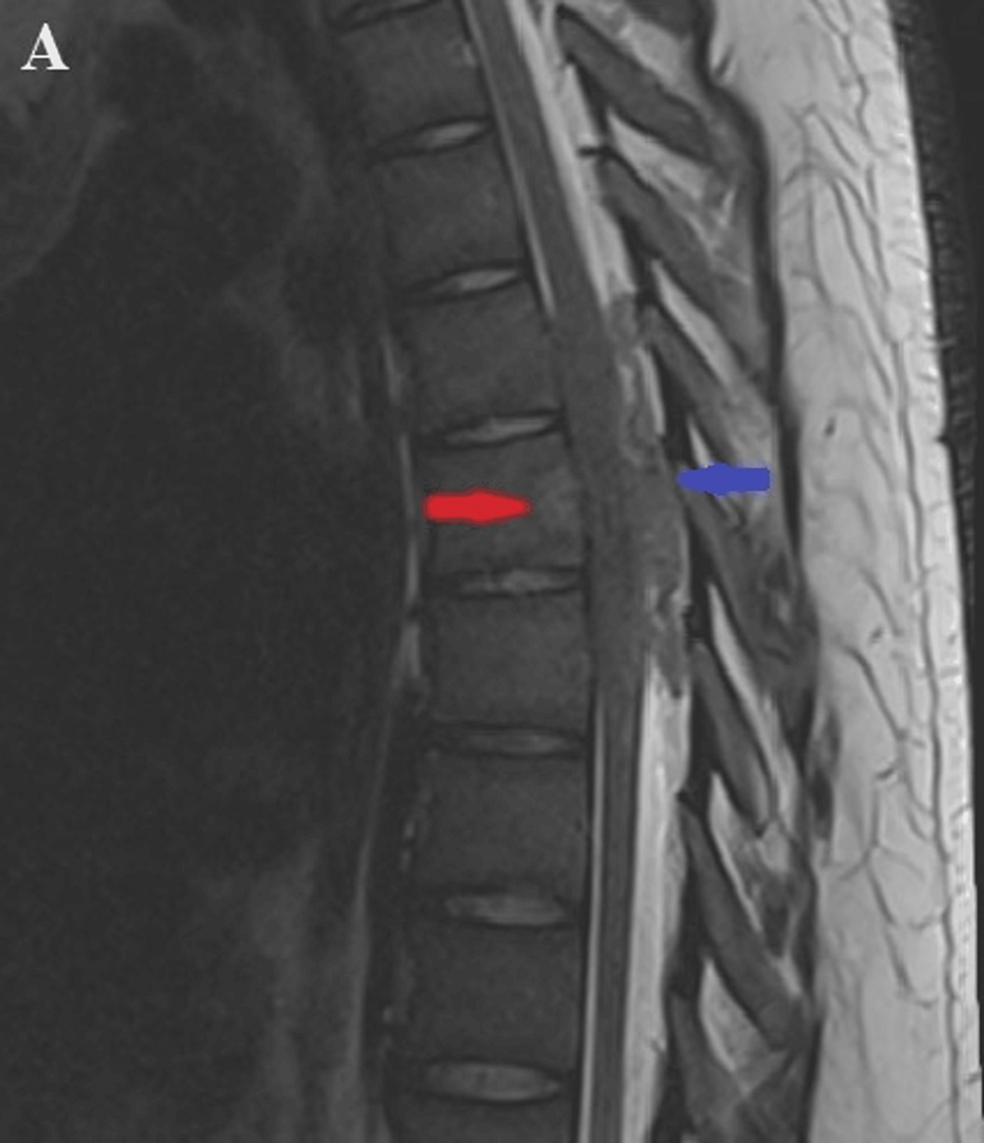
MRI of the thoracic spine in the sagittal and axial planes. The figure shows in T2 sequence isodense to hyperdense formation at the levels of TH5 to TH7 vertebral projections, a formation filling the posterolateral epidural space, compressing the adjacent spinal cord with local edema of the latter (blue arrow). Soft tissue destruction of the body of TH7 vertebra (red arrow).

**Figure 5:**
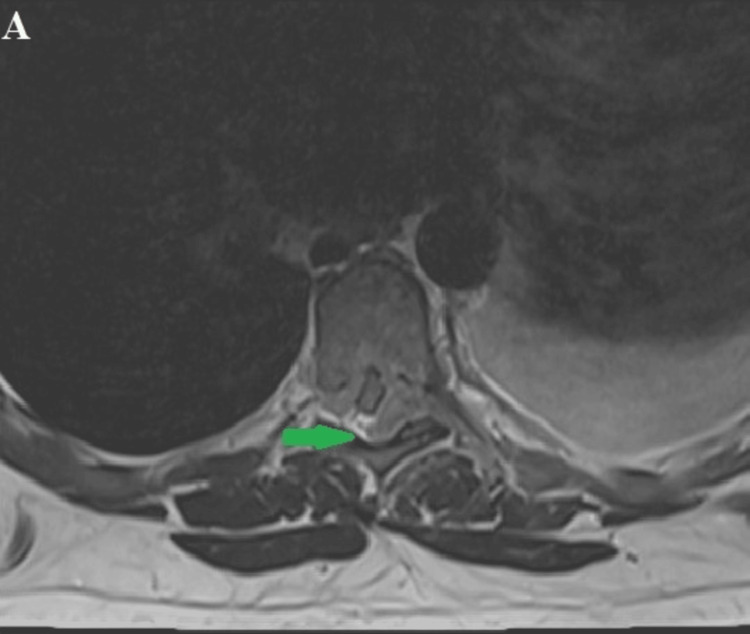
The figure shows an axial T2 sequence projection through the TH7 vertebral level showing a tumor mass (Ewing's sarcoma) as a hyperdense area located in the spinal canal and strongly compressing the adjacent medulla (green arrow).

**Figure 6:**
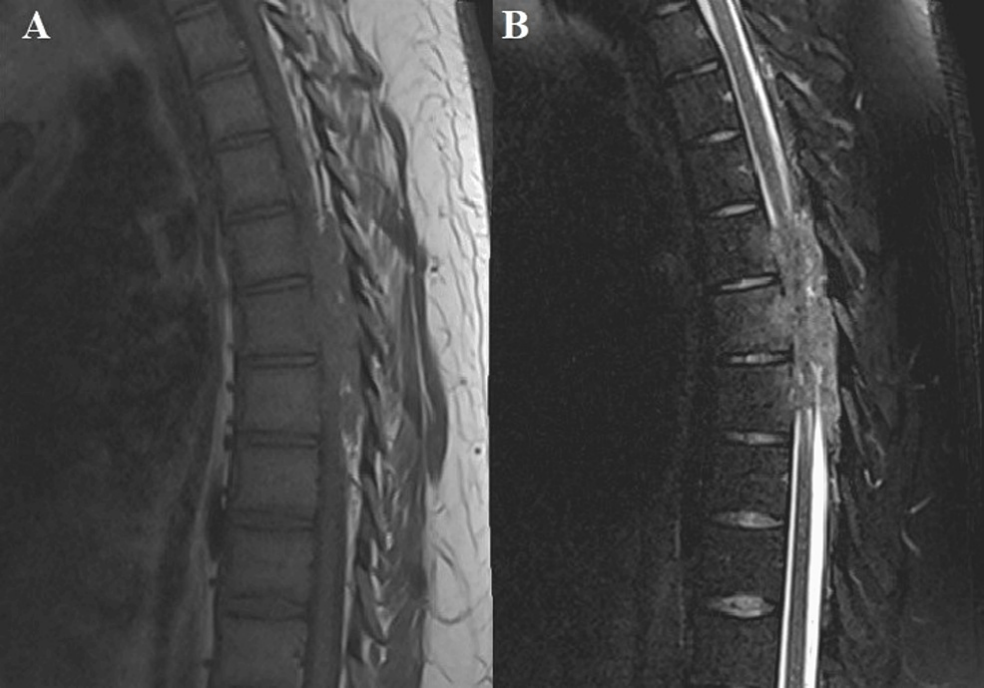
(A and B). MRI of the thoracic spine in T1 and T2 sequences in sagittal projections showing Ewing's sarcoma located from TH5 to TH7 vertebral projections and its relationship to the adjacent dura, as well as medullary compression. In Figure 6A on T1 sequence, the tumor has a hypointense density. In Figure 6B, Ewing's sarcoma is presented on a hyperintense T2 sequence.

A neurosurgical team, after reviewing the imaging and laboratory studies and clinically examining the patient, suggested the diagnosis of a malignant tumor in the thoracic region requiring surgical treatment. Based on the patient's young age, typical onset in the second decade of life, and the imaging findings, before the final histological result, we had a diagnostic doubt about the diagnosis of Ewing's sarcoma. An MRI of the cervicothoracic spine was recommended and subsequently performed, revealing at the TH5 to TH7 vertebral levels a hyperdense posterior epidural soft tissue mass with irregular structure and significant spinal cord compression in the T2 sequence. Additionally, a soft-tissue transformation of the TH7 vertebral body was identified (Figures 4-6).

Given the leading symptoms and supportive imaging findings, urgent surgical intervention was deemed necessary. The patient was transferred to the neurosurgery department, and the following day underwent surgery involving total laminectomy of the TH5, TH6, and TH7 vertebrae, excision of the soft tissue mass, and spinal cord decompression. A decision was made to perform a transpedicular stabilizing surgical intervention, if necessary, particularly in cases where there is a disruption of spinal biomechanics.

Postoperatively, the patient continued clinical recovery with individualized physical therapy, lasting for 14 days, during the hospital stay. He was discharged once he maintained an active posture while lying in bed and performed minimal movements of the lower extremities. Subsequent treatment was initiated, involving chemotherapy and radiotherapy at another oncology-focused hospital. The patient remained hospitalized for 17 days, during which his neurological status gradually improved with continuous monitoring and control measures.

Pathological finding

**Figure 7:**
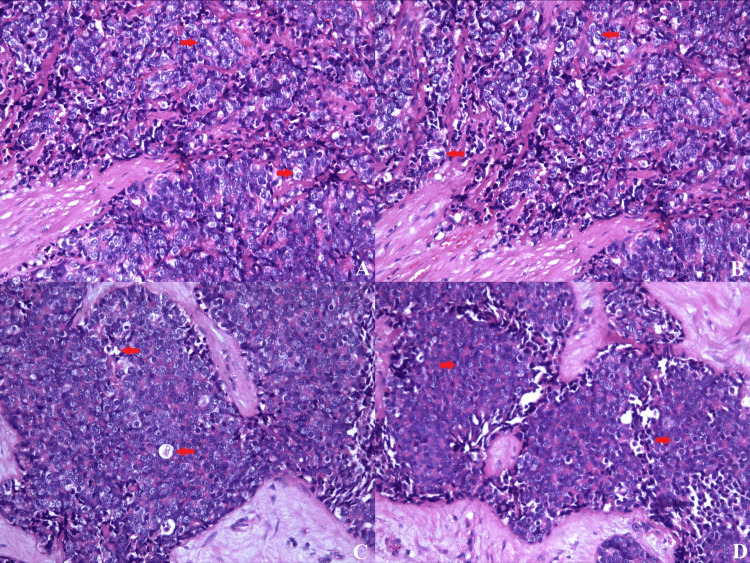
(A-D) Sheets of small, dark, round and uniform cells with scant cytoplasm, round nuclei, and small punctate nucleoli - typical of Ewing sarcoma (H&E stain; original magnification x100).

Macroscopic examination of the specimen showed fragmented, brownish material. Histologically, the tissue revealed tumor infiltration characterized by small, monomorphic cells with hyperchromic nuclei. The tumor presented solid areas of uniform, poorly differentiated small round cells with indistinct cell borders and finely distributed chromatin. Additionally, zones of necrosis and nuclear detritus were observed (Figure 7).

**Figure 8:**
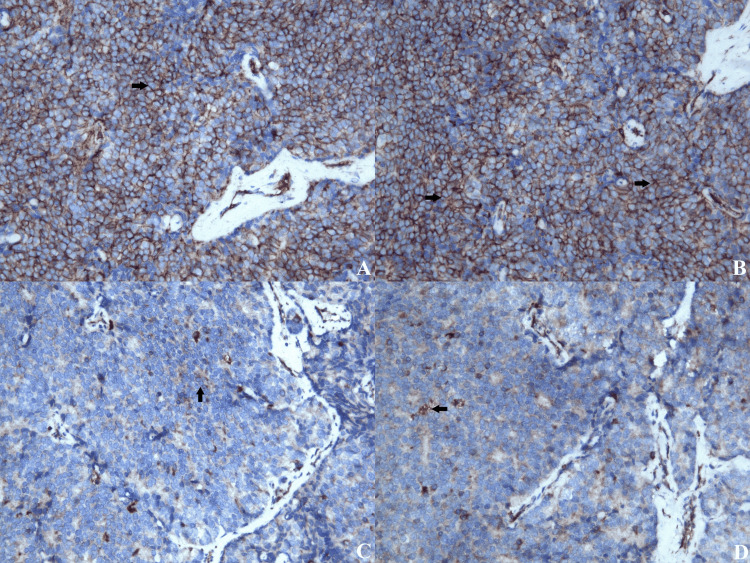
(A,B) CD-99 - Strong membranous diffuse staining in the tumor cells (H&E stain; original magnification x100). (C,D) S-100 - Weak heterogeneous expression pattern of S-100 protein (H&E stain; original magnification x100).

Immunohistochemistry testing showed that the tumor cells were negative for cytokeratin, TTF1, CD56, and NSE. More than 60% of the cells showed membrane positivity for CD99. There was also a weak focal expression of the S-100 protein (Figure 8).

These findings are consistent with a tumor from the Ewing Sarcoma Family of Tumors (ESFT), specifically Ewing Sarcoma. Testing for fusion genes EWS-FLI-1 or EWS-ERG is recommended.

Six months post-surgery, the patient is in an improved general condition and has achieved good oncological control without disease progression. Intensive physiotherapy for three months has led to significant recovery, with the patient regaining the ability to walk independently. Additionally, the pelvic-reservoir dysfunction has resolved, and normal urination has been restored.

## Discussion

Ewing's sarcoma is a malignant tumor that arises from neuroectodermal cells, histologically characterized by round cell sarcoma cells [4]. It was described for the first time in 1921 by James Ewing, with the most common population being patients around the second decade of life, i.e., its prevalence is among young people.

Microscopically, the tumor is characterized by clusters of small round blue cells with a sheet-like arrangement or pseudorosettes and sparse clear cytoplasm [1, 4]. From a differential diagnostic point of view, Ewing's sarcoma resembles lymphoma and rhabdomyosarcoma. For this reason, the expression of CD99, a membrane glycoprotein marker, serves as a key diagnostic tool in distinguishing between various tumor types. Some literature data indicate that this marker has high expression but lacks good specificity, necessitating the use of vimentin, S-100 protein, and synaptophysin [1, 2, 5, 6].

In our clinical case, CD99 examination revealed over 60% membrane positivity, supporting the diagnosis of Ewing's sarcoma. Additional studies supporting the diagnosis are the t(11;22)(q24;q12) and t(21:22) (q22:q12) translocations detected by molecular genetics [1, 7-10]. In our case, the indicated genes were not examined due to the lack of the necessary equipment.

Histological and immunohistochemical examination plays a major role in the diagnosis of Ewing's sarcoma. CT scan and MRI imaging have important guiding clinical value as well as a major role in planning surgical treatment. CAT gives information about the bone structures and the degree of their destruction when infiltrating the bone structures, as in our case. MRI has a good informative value regarding the contact of the tumor with the spinal cord, nerve roots and soft tissues. In T2-sequences, the tumor shows hyperintensity, and in T1 an isointense signal. Diffusion-weighted images (DWI) also provide good information, showing limited diffusion with a low value of the apparent diffusion coefficient [3, 7, 9]. In addition to the performed imaging studies of the spine, the native CT scan of the lung was also taken into account, where in our case a lesion located on the left in the area of ​​the 6th rib, along the middle axillary line, a soft tissue tumor formation, with destruction of the adjacent rib, was visualized which is assumed to be metastatic.

Literature indicates that for some cases of Ewing's sarcoma, biopsy followed by chemotherapy and radiotherapy is sufficient. However, due to the medullary compression and worsening neurological symptoms in this case, laminectomy was performed according to established neurosurgical principles. Histological analysis confirmed Ewing's sarcoma, with diffuse membranous CD99 expression in over 60% of the cells and weak focal expression of S-100. Additional markers CK, TTF1, CD56, and NSE were negative. Clinical pathology specialists recommended testing for fusion genes (EWS-FLI1 or EWS-ERG), but this could not be performed due to equipment limitations at the hospital.

It is difficult to differentiate, and there is controversy in the literature as to whether Ewing's tumor originates primarily from the white matter or whether the primary location is the thoracic spine and epidural space. Because of the rapid progression of this type of disease, we assumed that the primary tumor was located in the thoracic region or was combined with an Askin tumor that affected the chest. Due to the lack of additional research confirming or rejecting this scientific hypothesis, for the time being, the issue remains controversial and debatable.

We report a case of a mixed extraosseous and intraosseous form of the tumor characterized by cells similar to those classically described by Ewing. In 1969, a rare form of extraosseous type of tumor similar to Ewing's sarcoma was described. The prevalence of the latter is characteristic of the second and third decades of life and is equally prevalent in both men and women [11]. In our patient, a formation lysing the body of the TH7 vertebra was found, as well as the presence of an epidural part of the tumor, causing medullary compression and leading to residual neurological symptoms from the side of the lower limbs, as well as pelvic-reservoir disturbances. There are different opinions in the literature as to whether radical surgical treatment of this type of tumor leads to better patient survival. The combined application of surgical treatment, chemotherapy and radiotherapy was adopted after discussion by a multidisciplinary team [11].

Multiple studies have shown that in the absence of medullary compression, surgical biopsy is sufficient to establish the diagnosis. In our case, due to the present and progressively worsening neurological symptoms, as well as the presence of an epidural formation from Ewing's sarcoma proven by MRI, surgical decompression of the spinal cord was required. Based on Weinstein-Boriani-Biagini, on how much soft tissue, bony elements, and epidural space are affected, the surgical approach chosen was total laminectomy of TH5, TH6, and TH7, preserving the articulating joint complexes. In this way, medullary decompression was achieved, a large volume of the retrodural tumor formation was removed, and normal spinal cord function was enabled [12].

Literature data suggest that vertebral body infiltration can disrupt spinal column stability, warranting transpedicular stabilization and, if necessary, vertebrectomy and implant placement [13,14]. However, in our case, we determined that spinal stability was not significantly compromised at this treatment stage. Therefore, we opted to delay screw stabilization until after controlling tumor growth with chemotherapy and radiation therapy. Monitoring the patient up to the sixth-month post-surgery revealed no biomechanical disturbances in the thoracic spine.

According to the National Comprehensive Cancer Network (NCCN) guidelines, phase III clinical trials indicate that the optimal treatment approach involves preoperative radiotherapy or chemotherapy followed by surgery. However, in cases involving medullary compression, surgical intervention remains the primary treatment, followed by subsequent courses of chemotherapy [15]. One treatment option is the use of vinblastine, ifosfamide, doxorubicin, and etoposide, while another is the use of both adjuvant and neoadjuvant chemotherapy. Commonly administered medications include vinblastine, doxorubicin, and cyclophosphamide, with a cyclical regimen of ifosfamide and etoposide. These therapeutic strategies have demonstrated approximately 69% improvement in five-year survival rates [16].

## Conclusions

Ewing's sarcoma is a rare malignancy predominantly affecting individuals in their second decade of life. Typically, this tumor is found in the sacral region of the spine, followed by the lumbar and thoracic areas. The osseous form of the tumor is more common, often accompanied by the involvement of paravertebral soft tissues. This case highlights an epidural form of Ewing's sarcoma with both bony structure involvement and metastasis to the thorax.

The primary treatment approach involves surgical intervention, either through biopsy or maximal tumor excision, followed by chemotherapy and radiotherapy. Alternatively, initial tumor reduction can be achieved through adjuvant or neoadjuvant chemotherapy, with or without radiotherapy, as per established guidelines. In this particular case, due to significant spinal cord compression, the treatment began with surgical decompression, histological confirmation of the tumor, and subsequent chemotherapy and radiotherapy. Six months post-surgery, the patient has shown notable improvement, now mobilizing with assistive devices and continuing chemotherapy. Clinical and imaging studies indicate that spinal biomechanics remain intact.
